# Translation and cross-cultural adaptation of an Arabic version of PROMIS® of dyspnea activity motivation, requirement item pool and sleep-related impairments item bank

**DOI:** 10.1186/s12955-023-02223-w

**Published:** 2024-01-26

**Authors:** Monira I. Aldhahi, Hadeel R. Bakhsh, Bodor H. Bin Sheeha, Rehab Alhasani

**Affiliations:** Department of Rehabilitation Sciences, College of Health and Rehabilitation Sciences, Princess Nourah bint Abdulrahman University (PNU), Riyadh, P.O. Box 84428, 11671 Saudi Arabia

**Keywords:** Patient-reported outcomes, PROMIS, Dyspnea, Arabic, Sleep impairments, Physical function

## Abstract

**Background:**

Patient-Reported Outcomes Measurement Information System (PROMIS®) Dyspnea Activity Motivation & Requirement item pool and sleep related impairments (SRI) item bank are designed in assessing the impact of dyspnea and sleep and guiding patient management. However, to effectively utilize this tool in Arabic-speaking populations, it is essential to perform a thorough translation and cultural adaptation process. Therefore, the aim of the study is to translate and cross-culturally adapt the translated items of the PROMIS® dyspnea activity motivation and requirement and SRI into Arabic.

**Methods:**

A universal approach to translation adopted from PROMIS guideline document for translation and cultural adaptation, and the Chronic Illness Therapy translation methodology. The forward translation step followed by back work translation and Harmonization and quality assurance. Cognitive interview and pilot testing was conducted among 30 Arabic respondents across 5 different countries of Arabic speaker to produce a single version for Arab countries.

**Results:**

A successful translation and cross-cultural adaptation into Arabic was achieved while maintaining equivalency. The translation was clear and more colloquial sentences were semantically equivalent and easy to understand. Equivalence of meaning of PROMIS® dyspnea activity motivation, requirement and SRI were achieved. All items were appropriate, relevant to culture and it measured the same concept as the original items. In Items 2 of the dyspnea activity motivation related to leisure activity “shopping”, the term “catalog and website” was added instead of “catalog only” which makes item in line with the original source but more comprehensive and applicable to current shopping trends.

**Conclusions:**

The PROMIS® dyspnea activity motivation, requirement items pool and SRI item bank are culturally and linguistically suitable to be used in Arab country. By extending the accessibility of this measure to Arabic-speaking population, this study contributes significantly to the advancement of management and patient-centered care in the region. Further studies are necessary to evaluate the psychometric properties of these instruments.

**Supplementary Information:**

The online version contains supplementary material available at 10.1186/s12955-023-02223-w.

## Background

Patient-reported outcomes (PROs) play a pivotal role in modern healthcare as they provide a direct assessment of a patient's health status, symptoms, functional ability, and quality of life [1]. Patient-reported Outcomes Measurement Information System (PROMIS®) tools have emerged as valuable instruments for assessing various aspects of health-related quality of life, providing standardized measures that transcend cultural and linguistic barriers [2, 3]. PROMIS® is initiative developed by National Institution of Health that are used for clinical research and practice and stands as a leading system for PROs measurement, providing standardized, reliable, and valid measures across diverse health domains [3]. Among the domains of PROMIS are dyspnea and sleep impairments [4], which hold significant importance in clinical and research settings due to their profound impact on the overall well-being and functionality of individuals.

Dyspnea, a distressing symptom characterized by a subjective perception of difficulty breathing, affects individuals across various medical conditions and has significant implications for individual's quality of life [5]. Its multidimensional nature encompasses physical, emotional, and social components, making it imperative to comprehensively assess its impact on individuals' daily lives [6]. Dyspnea significantly influences individuals' activity motivation and requirements, affecting their ability to engage in physical activities, limiting participation in social interactions, and impacting overall health-related quality of life [7, 8]. As with dyspnea, sleep plays a pivotal role in individuals' health and well-being. Disrupted sleep patterns is prevalent in Saudi Arabia [9] leading to a myriad of adverse health outcomes, such as reduced cognitive function, and an increased risk of chronic health conditions [10–12]. Therefore, accurately evaluating sleep-related impairments, as well as dyspnea activity motivation and requirements, is essential for comprehending their impact on functional abilities, and overall well-being. It is crucial in providing comprehensive care and developing targeted interventions to improve individuals sleep quality and overall health outcomes.

PROMIS® has garnered considerable attention for its comprehensive and psychometrically robust assessment of PROs [3, 4]. This standardized measurement system has revolutionized the assessment of PROs, as it provides researchers and clinicians with a common metric to evaluate various health domains across different patient populations [4, 13]. It is a set of person-centered measures used to determine rehabilitation interventions' effectiveness and evaluate change over time, to predict the future outcome of interventions or diagnoses. PROMIS of Dyspnea Activity Motivation, Requirement items and sleep related impairment (SRI) item bank are widely used instruments designed to assess the impact of dyspnea and sleep on activity-related motivations and requirements [14, 15].

The PROMIS® Dyspnea Activity Motivation & Requirement pool focuses on assessing the impact of dyspnea on activity-related motivations and requirements. While the PROMIS SRI intended to measure self-reported levels of wakefulness, sleepiness, fatigue, and functional limitations associated with sleep issues during the past seven days [14]. The PROMIS Dyspnea Activity Motivation & Requirement and sleep-related impairment provide a unique lens into the patient experience, capturing not only the functional limitations imposed by dyspnea and sleep pattern but also the motivation and drive to engage in daily activities despite its challenges. The measure delves into the psychological aspects of dyspnea management, shedding light on individuals’ resilience and coping mechanisms.

The process of translating and cross-culturally adapting the PROMIS three item banks represents a critical step toward enabling a comprehensive assessment of dyspnea and sleep in Arabic-speaking individuals. However, we lack the Arabic translation of the item which dependence on adhering to internationally recognized guidelines and methodological rigor. To fully leverage the benefits of this measure in Arabic-speaking populations, a rigorous process of translation and cross-cultural adaptation is required. The importance of cross-cultural adaptation of PROMIS of dyspnea activity motivation, requirement and SRI item banks lies in the recognition of cultural diversity and linguistic nuances in different populations [16].Therefore, the overarching aim of the study is to translate PROMIS® Dyspnea Activity Motivation & Requirement pool, and Sleep Impairment item bank into Universal Arabic language and to culturally adapt the translated version for implementation in clinical settings. Therefore, we aim to provide a robust and culturally sensitive instrument for assessing dyspnea activity motivation and activity requirement and dyspnea-related impairments in Arabic-speaking population. Lastly, the outcomes of this study would contribute to the availability of standardized assessment tools for dyspnea and sleep management in Arabic-speaking populations.

## Methods

### Study design and translation team

We conducted a cross-sectional methodological study between April 2022- Feb 2023. The authors obtained the required licenses and authorization from the PROMIS Health Organization (PHO) to translate the following: PROMIS Dyspnea Activity Motivation & Requirement Pool and SRI item bank. The translation team were a panel of experts composed of healthcare providers and language professionals and native Arabic-speakers from Saudi Arabia, Egypt, Jordan, Lebanon, Palestine, and Syria (Additional file 1). All linguists included in the translation team comply with ISO 17100 standards for professional competencies and translator qualifications.

### Participants

An iterative process of translation for the harmonization included Arabic-speaking adult respondents from different countries (Egypt, Jordan, Kuwait, Morocco, and Saudi Arabia) were included in the study to assess the relevance, comprehensibility, and appropriateness of the translations and adaptation through cognitive interviews conducted in Arabic. We have included adults’ individuals age between 18- 65 years irrespective of sex, who were a native Arabic speaker with diverse educational backgrounds who have no prior history of psychological or mental health issues. Patients with a history of severe medical conditions, such as chronic kidney disease, chronic liver disease, psychiatric disorders, and malignancies, were excluded from the study. Participants selected through a convenient sampling method were surveyed to collect their perspective on the questionnaire. Those participants who declined to participate in the study or were unable to read Arabic were excluded from this study. The qualitative assessment of cognitive interviews aided in establishing the linguistic equivalence of each translation and provided insight into the significance of the concepts. The study protocol was approved by IRB Ethical Committee of Princess Nourah bint Abdulrahman University (PNU 22–167) in Riyadh, Kingdom of Saudi Arabia, and all of the participants provided informed consent.

## Outcome measures

The items pool of the PROMIS® Dyspnea Activity Motivation assess individuals' drive and motivation to maintain their activity levels. This item pool encompasses of seven items that inquire about participants' inclination to engage in various daily activities. The PROMIS® Dyspnea Activity Requirements is consists of four items that evaluate the influence of an individual's surroundings on their physical activity levels. PROMIS® sleep-related impairments item bank is 16 items designed to provide valuable insights into an individual's sleep quality and overall well-being. All items rely on seven-day recall and a Likert-like scale, ranging from 1 (not at all) to 5 (very much).

### Translation and culture adaptation process

The translation was conducted in line with the PROMIS® guideline document for translation and cultural adaptation, and the Chronic Illness Therapy (FACIT) translation methodology which comply with the international guidelines by ISPOR (The Professional Society for Health Economics and Outcomes Research) [17, 18]. The translation procedure was completed in collaboration with members of the PROMIS® group, and items translated from English to Arabic applying the FACIT Measurement procedure (www.facit.org). This translation method is designed to address the three dimensions of cross-cultural equivalence of meaning: semantic (the same meaning), cultural (culturally appropriate), and conceptual (measuring the same theoretical construct) [19].

**Fig. 1:**
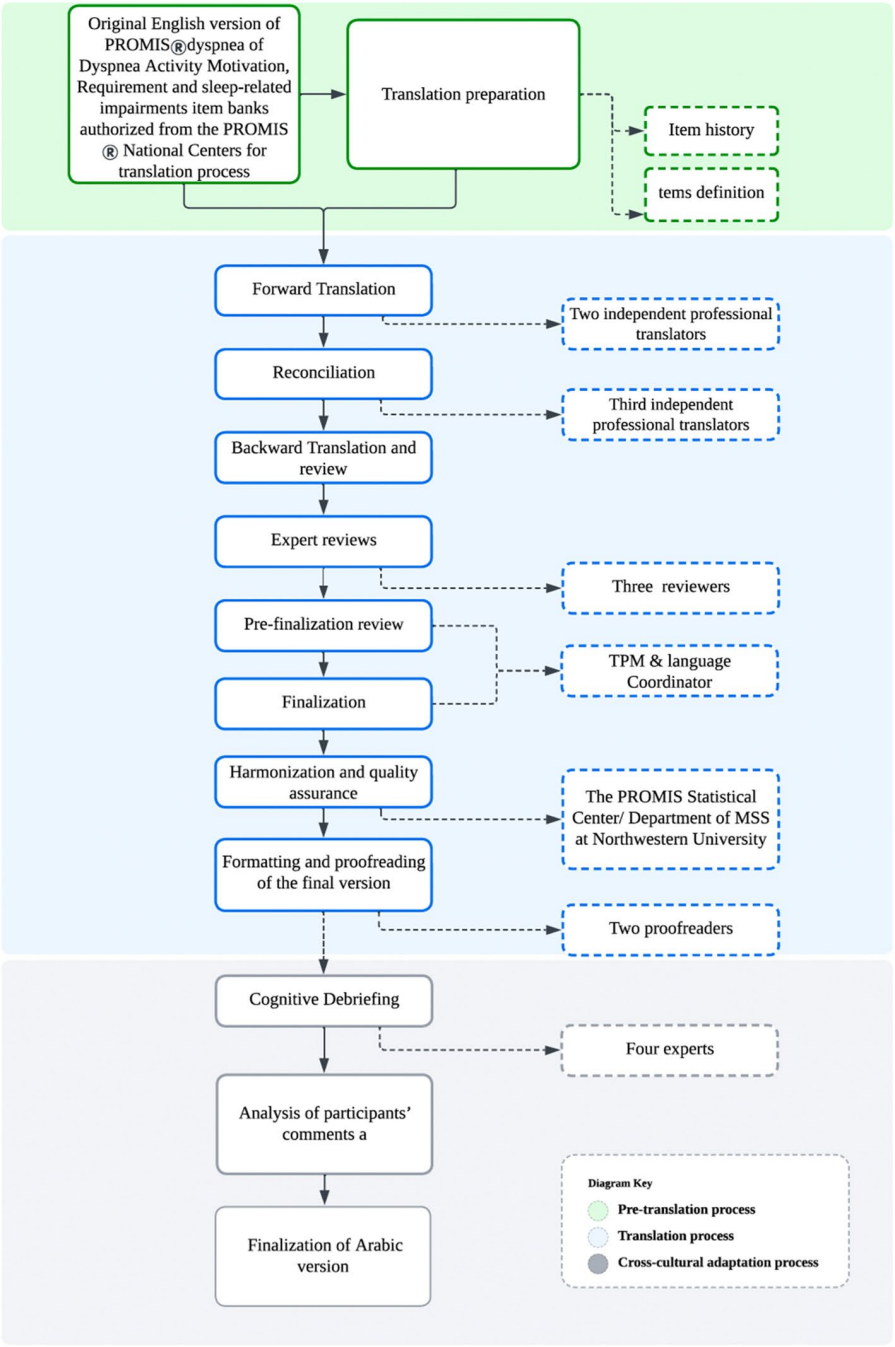
Graphic illustration of the stages of the translation and cross-cultural adaptation

The following steps outline the FACIT translation process (Fig. 1):

1. *Preparation of the translation documentation (Item History) and item definitions*: In this process the items are included in an Item History document, where each item, its translations, and related comments are listed on separate columns of an Excel document. This format allows for a focused approach to each translation item, facilitating visual comparison of different translations and back-translation, as well as providing a platform for translators and reviewers to offer comments on each item. Subsequently, the final translated version of each item is then formatted according to the project's requirements for the pre-testing phase and subsequent distribution. In the process of item definition, FACITtrans and the Department of Medical Social Sciences (MSS) at Northwestern University’s (NU) Feinberg School of Medicine created a document that includes an explanation of the concept evaluated in each item, along with the technical definition of each term used in the item. This document is intended to act as a reference for the Translation Project Manager (TPM) and all translators involved in the translation process.
2. *Forward Translation*: The initial step involves the translation of the original PROMIS®Dyspnea Activity Motivation & Requirement pool into Arabic by a team of bilingual experts. The forward translation process aims to capture the intended meaning of each item while maintaining linguistic accuracy. The translator deemed to have a deep understanding of both the source language (English) and the Arabic language to accurately capture the conceptual meaning of each item.
3. *Reconciliation*: The forward-translated version is then reviewed and reconciled by the third translator who is a native speaker of the Arabic language. The translator compares the forward-translated version to the original instrument to ensure that the intended meaning of each item is retained and culturally appropriate for the target population. This achieved through the selection of a single forward translation or the creation of a hybrid version by combining the two translations.
4. *Backward Translation*: An independent translator fluent in the Arabic language and native English speaker who is blinded to the original instrument, was asked to assess conceptual equivalence and identify potential discrepancies. By engaging bilingual experts, we ensure that the translation process is rigorous and maintains fidelity to the original instrument.
5. *Back-translation review*: A native English speaking fluent in Arabic compares source and back-translated English versions to identify discrepancies in the back-translations. This step also leads to an initial assessment of the harmonization between the languages.
6. *Expert reviews*: Three native speakers of the Arabic language, who are experts in the field, independently review all of the preceding steps and determine the most suitable translation for each item or suggest alternative translations if the previous ones are not acceptable
7. *Pre-finalization review*: The TPM evaluates the usefulness of the reviewer's feedback, pinpoints probable difficulties in their recommended translations, and formulates queries and remarks to assist the Language Coordinator (LC)for the target language.
8. *Finalization*: The LC who should possess native fluency in the Arabic language, determines the ultimate translation by thoroughly examining all the information presented in the Item History and addressing the comments made by the TPM. In addition to the final translation, the LC also provides both the corresponding literal back-translation and refined back-translation for each item.
9. *Harmonization and quality assurance*: The assessment of the accuracy and equivalence of the final translation carried out by the Translation Project Manager, who compared the final back-translations with the source text and ensured that comprehensive documentation of the decision-making process is in place. The PROMIS® Statistical Center/The Department of MSS at NU Feinberg School of Medicine was included in these steps of the translation process. A quality review includes checking for consistency with previous translations and other languages if relevant, as well as ensuring consistency among the items.
10. *Formatting and proofreading of the final version*: The translated items in the item history were transferred to the Excel file formats in which two proofreaders work independently, and reconciliation of the proofreading comments. Any modifications made to the questions during proofreading are recorded in the Item History. This ensures that the most recent version of the translated items is always documented.
11. *Cognitive Debriefing*: An interview script template is created and translated into the Arabic language. Cognitive debriefing interviews were conducted in a structured interview format by the authors with a sample of a total of 30 participants who are native Arabic speakers from five Arabic countries. Each item of the questionnaire is debriefed with 6 participants from each country. Each participant is instructed to first respond to the items independently. After completing the questionnaire, a cognitive debriefing interview is conducted. This interview involves a bilingual interviewer who poses some general questions to the participant, aiming to obtain feedback on the difficulty level of any items and to determine whether any items are offensive or irrelevant. This is followed by inquiries concerning the participant's comprehension of the items. During the interview session, the interviewer and participant collaboratively reviewed the participant's responses. Participants are encouraged to voice any difficulties encountered while responding to the items, and their feedback is invaluable in refining the translation and ensuring its cultural relevance. The template used in the interview contains a series of inquiries, including: identification of the selected item and the rationale behind the choice; eaboration on the interpretation or conceptual understanding of the chosen item; assessment of the clarity and ease of understanding of the provided options; evaluation of the logical order of the options; if found illogical, suggestions for alternative arrangements are invited; Personal elucidation of the item's meaning; justification for the chosen response; explanation of the respondent's understanding of the accompanying instructions; Identification of any items that were unclear or led to confusion, with accompanying explanations. Additionally, in certain instances, two options with equivalent meanings are presented, and respondents are encouraged to provide suggestions on which term they believe is more appropriate and readily comprehensible. This questionnaire aims to gather comprehensive data by probing participants on their item choices, comprehension of options, potential areas of ambiguity, and recommendations for improvement. The data collected from these interviews are carefully analyzed to identify patterns, themes, and areas for improvement in the translated measure.
12. *Finalization of Arabic version*: The feedback from cognitive debriefing interviews compiled by the TPM and back-translated into English and summarize the issue with feedback. The LC goes through the issues and suggests an alternative option. The TPM ensures that the solutions proposed by the align with the source and other languages. This Arabic version is considered culturally and linguistically equivalent to the original English instrument, allowing for meaningful and reliable assessments in the population.

## Results

A total of 30 Arabic-speaking adult respondents from Egypt, Jordan, Kuwait, Morocco, and Saudi Arabia completed the item pool and the item bank through cognitive interviews conducted in Arabic. The debriefed participants' characteristics reflected a balanced representation of gender, with 15 males and 15 females, resulting in a ratio of approximately 1:1. The participants' mean age was 35 ± 11 years, and the majority (*n* = 12, 40%) had attained bachelor's degrees as their highest education level. The findings of the study indicated that 36.67% (*n* = 11) of the respondents had a high school degree, while 23.33% (*n* = 12) of the participants held professional degrees or literary certificates.

The debriefed participants provided explanations that corresponded well with their answer choices. Participants reported that the questions were clear; in 100% of items, 100% of the instructions and 85.71% of the response formats. Items of dyspnea activity motivation (DYSAM001 and DYSAM003 through 7) were straightforward and did not require any major changes after the first step. However, the response format of DYSAM002 was adjusted at later stages of the procedure, indicating the careful attention given to ensure linguistic and cultural equivalence. In the context of DYSAM002, where participants are prompted to select a preferred activity, one of the choices presented was “shop for an item through a catalogue.” A majority of the respondents expressed consensus that the term “catalogue” alone does not sufficiently encompass modern technological advancements, particularly in the realm of online shopping. They argued that the term “catalogue” does not adequately convey the concept of shopping online. Therefore, respondents suggested that the statement could be improved by incorporating the phrase “shop for an item through a catalogue or a website,” as it would accurately capture the contemporary practice of online shopping. In Arabic, this enhancement would be expressed as “التسوّق لشراء منتج من خلال كاتالوج أو موقع إلكترونيّ”. Table 1 represents a summary of the conclusions derived from the process of translation related to one of the items that require minor modification.

**Table 1:** Example of the key issues raised in the process of the finalization of the dyspnea PROMIS® pool item activity motivation

PROMIS® item code	DYSAM002	
**Tested Item stem**	English version:	Arabic version:
	Imagine that you had no problem with shortness of breath, and that you had time to do what you wanted to do. Please indicate below which activity you would generally prefer to do, assuming you had the ability to do both equally.	تخيّل لو لم تكن لديك مشكلة في ضيق التنفّس، ولو كان لديك الوقت لتفعل ما تريد القيام به. يرجى الإشارة أدناه إلى النشاط الذي تفضل القيام به بشكل عام، بافتراض أن لديك القدرة على القيام بالأمرين بشكل متساوي.
**Item responses and score**	0 = Shop for an item through a catalogue 1 = Shop for an item by walking through a store	0=التسوّق لشراء منتج من خلال كاتالوج 1=التسوّق لشراء منتج من خلال التجوّل في المتجر
**LC Response**	The participant’s suggestion is to rectify one of the options and add “through a website” instead of only “catalogue.” 0 = Shop for an item through a catalogue or a website 1 = Shop for an item by walking through a store	
**FACIT and MSS Response**	Please include both options: “catalogue or website”. This way the item still is in line with the source but also more applicable to current shopping trends	
**Post Test Final**	0 = Shop for an item through a catalogue or a website 1 = Shop for an item by walking through a store	= 0 التسوّق لشراء منتج من خلال كاتالوج أو موقع إلكترونيّ = 1 التسوّق لشراء منتج من خلال التجوّل في المتجر

*Abbreviations*: *FACIT* Chronic Illness Therapy translation, *MSS* Department of Medical Social Sciences, *LC* Language coordinator

Regarding the Dyspnea Activities Requirements. The debriefed participants provided explanations that corresponded well with their answer choices. Participants reported that the questions were straightforward in terms of concept 75% of items, 75% in the questions wording usage, 100% of the instructions and 100% of the response formats. Only item three (DYSAR003) was identified as having an issue in terms of the Arabic term for “stairs” with a suggested alternative of “الدرج” instead of “السلالم” However, based on expert input, it was determined that the current translation had been thoroughly tested and validated in previous physical functioning measures and did not present any issues. Therefore, no data supported a change, and the suggestion was deemed preferential, resulting in the retention of the original translation (Table 2).

**Table 2:** Example of the key issues raised in the process of the finalization of the dyspnea PROMIS® pool item activity requirement

PROMIS® item code	DYSAR003	
**Tested Item stem**	English version:	Arabic version:
	In the past month, have you moved into a new home or place that requires fewer trips up or down stairs?	خلال الشهر الماضي، هل انتقلت إلى منزل أو مكان جديد يتطلّب عدداً أقلّ من مرات الصعود والهبوط على السلالم؟
**Item responses and score**	0 = No 1 = Yes	0 = لا 1 = نعم
**LC Response**	The five debriefed participants provided explanations that corresponded well with their answer choices. The client suggested a change to the word for “stairs” Test version: هل انتقلت إلى منزل أو مكان جديد يتطلّب عدداً أقلّ من مرات الصعود والهبوط على **السلالم**؟ Suggested wording changes version: هل انتقلت إلى منزل أو مكان جديد يتطلّب عدد أقلّ من مرات الصعود والهبوط على **الدرج**؟	
**FACIT and MSS Response**	The suggested change is to the word “stairs”. A decision was made previously to use الدرج. Based on MSS feedback, throughout the validation of physical function measures, they had extensive discussions on the chosen translation for “stairs.” The current translation has been thoroughly tested, validated, and does not present issue. Since there is no data to support a change and the suggestion is preference. No translation changes are recommended	
**Post Test Final**	In the past month, have you moved into a new home or place that requires fewer trips up or down stairs?	خلال الشهر الماضي، هل انتقلت إلى منزل أو مكان جديد يتطلّب عدداً أقلّ من مرات الصعود والهبوط على السلالم؟

*Abbreviations*: *FACIT* Chronic Illness Therapy translation, *MSS* Department of Medical Social Sciences, *LC* Language coordinator

After the cognitive debriefing process, the participants shared their insights while responding to the SRI item banks, demonstrating a thorough comprehension of its content. Their responses indicate a clear and accurate understanding of the item's intent. Participants reported that all items were clear in terms of sematic and cultural appropriate and 93.75% of the items in the questions wording usage (one changes in the item Sleep 123 (Table 3)),100% of the instructions and 100% of the response formats. The final linguistically Arabic version can be requested from https://www.healthmeasures.net/.

**Table 3:** Example of the key issues raised in the process of the finalization of the sleep-related impairments item banks

PROMIS® item code	Sleep123	
**Tested Item stem**	English version:	Arabic version:
	I had difficulty waking up	كنت أجد صعوبةً في الاستيقاظ
**Item responses and score**	1 = Not at all 2 = A little bit 3 = Somewhat 4 = Quite a bit 5 = Very much	1 = ليس على الإطلاق 2 = مرات قليلة 3 = نوعاً ما 4 = غالبا 5 = كثيراً جداً
**LC Response**	The participants suggest revising the item from “كنت أجد صعوبة في الاستيقاظ” to “كنت أجد صعوبة في الاستيقاظ **من النوم**” The difference is adding “from sleeping” at the end of the item this made the sentence more understandable	
**FACIT and MSS Response**	Based on the participant suggestion the LC agrees with the change, but note that no participant had any issue with the test version translation. MSS agreed one can only “wake-up” from sleep. However, some dialects do not use the current word in spoken language. “from sleep” clarifies the meaning in the case of those with lower education levels who may not recognize the current term	
**Post Test Final**	I had difficulty waking up	كنت أجد صعوبةً في الاستيقاظ من النوم

*Abbreviations*: *FACIT* Chronic Illness Therapy translation, *MSS* Department of Medical Social Sciences, *LC* Language coordinator

## Discussion

This study presents the process of translating and cross-culturally adapting the PROMIS of two item pool of Dyspnea Activity Motivation & Requirement into Arabic and the item bank of the SRI. The translation and cross-cultural adaptation process underwent rigorous testing to ensure the linguistic and cultural equivalence of the Arabic version. Cognitive interviews played a pivotal role in confirming the questionnaire's clarity and relevance to the participants, further validating the adapted instrument. The findings suggest that the Arabic versions of the Dyspnea Activity Motivation & Requirement item pool and SRI item bank are suitable for use in Arabic-speaking populations. We had good agreement between the translators and reviewers for most of the items. The high clarity and understanding demonstrated by the participants indicate the successful translation and adaptation process. The retention of the original translation for item underscores the importance of considering expert input and previous validation to maintain the instrument's integrity.

Healthcare professionals encounter a diverse patient population, including those from various cultural backgrounds and language preferences. The language and cultural barriers have limited the widespread use of standardized measures like the PROMIS of Dyspnea Activity Motivation, Requirement and SRI. Furthermore, to improve the health-related functioning of patients or clients effectively, it is crucial to comprehend individual factors, such as activity related requirements and motivation. The PROMIS® Dyspnea Activity Motivation and Requirement tool offers a comprehensive approach that facilitates a deeper understanding of the patient's experience and functioning, particularly within the Arabic-speaking population. The most commonly used PROMIS dyspnea item banks primarily focused on addressing dyspnea sensation and its emotional response [20]. Yet, it is required that adapt other tools to assess contextual factor related to motivation and activity. Previously, the validity of these items was established within the Swedish population [21]. Therefore, this study addresses this limitation by providing an Arabic version of the PROMIS instrument, facilitating its adoption in clinical practice and research is required. Thus, ROMIS ® Dyspnea Activity Motivation and Requirement tool are typically brief and efficient, reducing respondent burden while still providing robust data for analysis.

In addition, the sleep-related impairments item banks related to sleep impairments have seen widespread use and have been translated into multiple languages and applied in diverse populations [13, 22–24]. Given the high prevalence of these items underscores a compelling justification for their translation into Arabic and their subsequent application within this specific context. Therefore, the availability of an Arabic version of the PROMIS of Dyspnea Activity Motivation & Requirement and SRI are of paramount importance in ensuring equitable healthcare delivery and enhancing the validity of clinical research conducted in the region. Such adaptations ensure that the instrument maintains its conceptual equivalence and relevance, allowing for accurate and culturally sensitive assessments.

The translation and cross-cultural adaptation process carries implications beyond mere linguistic conversion. It contributes to the acknowledgment and understanding of cultural variations in the perception and expression of dyspnea and its associated challenges. By embracing these cultural nuances, healthcare professionals can provide more patient-centered care, tailored to the unique needs and preferences of the individuals they serve. Moreover, the availability of a culturally adapted version of the PROMIS of Dyspnea Activity Motivation & Requirement and SRI promotes standardized assessments, facilitating the comparison of dyspnea and sleep-related outcomes across different populations.

In a recent scoping review highlighted the necessity for the importance of a rigorous methodological approach that address cross-cultural adaptation of patient-reported outcome measures in Arabic speaking countries [25]. Furthermore, a systematic review that focuses on PROMs within Arabic-speaking populations provide an overview of the status and challenges related to PROMs in the linguistic and cultural context. The review noted the absence of information on the translation process in certain studies and recommended enhancements in methodological aspects for future research [16]. Further strength of the study is that we have implemented standard best practice translation guidelines by involving translators and reviewers from multiple countries in accordance with the FACIT guidelines. One advantageous aspect of the FACIT guidelines is its emphasis on the universal translation approach. This facilitates the translations to be more applicable for individuals speaking the target language across multiple countries [19]. Therefore, through this study we ensure the complete translation and cultural adaptation of the adult PROMIS SRI and dyspnea items into Arabic. This allows us to take the next step and conduct psychometric testing among the Arabic population [24–26]. The study's outcomes hold considerable implications for clinicians, researchers, and policymakers in the field of dyspnea and sleep management, offering a standardized tool to inform patient care and improve the overall well-being of individuals with dyspnea. Furthermore, the availability of a culturally adapted instrument ensures equitable access to accurate assessments, fostering patient-centered care and supporting cross-cultural research.

While this study possesses several strengths, it is essential to acknowledge certain limitations. The study included participants with various educational backgrounds and healthy population, it did not specifically target specific clinical populations. Therefore, the findings' generalizability to these particular populations remains uncertain. Future research should aim to incorporate diverse clinical populations and individuals to ensure the broader applicability of the translated items. We would like to acknowledge the difficulty in eliminating variations between literary and spoken Arabic dialects, as well as differences in vocabulary among Arabic speakers in Western countries. Therefore, it is imperative to recognize the necessity for tailored translation and adaptation of instruments to specific cultural contexts in some Arabic-speaking countries.

## Conclusion

In conclusion, the translation and cross-cultural adaptation of the adult PROMIS Dyspnea Activity Motivation & Requirement item pool and SRI item bank into Arabic provides unique insights into individuals functional limitations and the psychological impact of dyspnea and sleep deficiency. Cultural adaptation ensures that the instrument captures the subtleties of language and cultural norms. The availability of a standardized and culturally relevant measure empowers healthcare professionals to provide patient-centered care and fosters cross-cultural research efforts in dyspnea management. Ultimately, the translated PROMIS measure represents a significant step towards enhancing patient care and understanding the full impact of dyspnea on individual’s lives. Future study is required to assess the psychometric properties and establish the reliability, validity, and measurement properties of the translated Arabic items.

### Supplementary Information

**Additional file 1.** Background of the role and responsibility of the translation team.

## Data Availability

The identified datasets analyzed during the current study are available from the corresponding author on reasonable request.
