# Correction: Translation and cross-cultural adaptation of an Arabic version of PROMIS® of dyspnea activity motivation, requirement item pool and sleep-related impairments item bank

**DOI:** 10.1186/s12955-024-02240-3

**Published:** 2024-03-12

**Authors:** Monira I. Aldhahi, Hadeel R. Bakhsh, Bodor H. Bin Sheeha, Rehab Alhasani

**Affiliations:** Department of Rehabilitation Sciences, College of Health and Rehabilitation Sciences, Princess Nourah Bint Abdulrahman University (PNU), P.O. Box 84428, 11671 Riyadh, Saudi Arabia

**Correction: Health Qual Life Outcomes 22, 11 (2024)**

**https://doi.org/10.1186/s12955-023-02223-w**

The authors of the original article [1] wish to clarify with respect to Corresponding Authorship that Monira I. Aldhahi's role was limited to that of paying and proofing author during production, whereas Rehab Alhasani's role is that of primary Corresponding Author nominated to handle all queries post-publication.
