# Supplementary material for: Translation and cross-cultural adaptation of an Arabic version of PROMIS® of dyspnea activity motivation, requirement item pool and sleep-related impairments item bank
Source: Health Qual Life Outcomes. 2024 Jan 26;22:11. doi: 10.1186/s12955-023-02223-w (PMC10821257; doi:10.1186/s12955-023-02223-w)
Supplement: Supplementary file 1 — Additional file 1. Background of the role and responsibility of the translation team. [file 12955_2023_2223_MOESM1_ESM.docx]

| **Additional file 1. Background of the role and responsibility of the translation team** | | |
| --- | --- | --- |
| **Role** | **Education Background** | **Title & Profession** |
| Translation Project Manager  FACITtrans (TPM) | BA, Linguistics | Senior clinical Outcome Assessments Translation Project Manager – Life Sciences |
| Forward 1 | BA, Languages and Translation, Simultaneous Interpretation (English & Arabic) | Senior Translator, Copywriter & Proofreader  Professional and Translator Interpreter |
| Forward 2 | MA, Linguistics | Professional Translator and Interpreter |
| Reconciler/Proofreader | Ph.D., Linguistics | Professional Linguist and Translator |
| Back Translator | MA, Diplomacy  BA, Medical Technology | Professional Translator  16 years full immersion in Arabic public school system and 3.5 years of undergraduate education (nursing) at King Abdul Aziz University, Jeddah Saudi Arabia |
| Reviewer 1 | Ph.D., Linguistics | Linguist and researcher  Pragmatics, sociolinguistics, discourse analysis, ideology, identity and translation studies |
| Reviewer 2 | Ph.D., Linguistics | Professional Linguist and Translator |
| Reviewer 3,  Language Coordinator, Proofreader 1 | DDS  MA, Biblical Studies | Professional Translator and Interpreter  Close to 30 years’ experience specializing in medical, legal and religious translation |
| Focus group interviewers | Ph.D., Rehabilitation Science | Professional clinicians with more than 7 years experiences in Rehabilitation Sciences and patient outcome measure. |
